# Encephaloid Tumor of the Antrum—Excision of Upper Jaw—Recovery

**Published:** 1872-10

**Authors:** Hunter M'Guire


### ARTICLE IX.

### *Encephaloid Tumor of the Antrum—Excision of Upper Jaw—Recovery.*

BY PROF. HUNTER M'GUIRE, M. D.

Pleasant Jones, aged 57 years, from Raleigh, N. C., was admitted into the College Infirmary, May 6th, 1872, with a large encephaloid growth of the upper jaw. The tumor began in the posterior wall of the antrum six or eight months before, increased slowly for four months, when it commenced to grow very rapidly. The tumor seen upon the jaw was about the size of a man's fist, distended the nostril of that side, and a portion about the size of a hen's egg protruded into the mouth. It was very painful, offensive, bleeding sometimes spontaneously, and always when handled even in the gentlest and most careful manner. After a day or two preparatory treatment, Dr. McGuire decided to remove the upper jaw. Chloroform having been given, the incision recommended by Sir William Ferguson was adopted. The upper lip was divided in the middle line, the left nostril laid open, and the knife carried along the side of the nose as far as the nasal bone. The knife was then passed horizontally across the lower border of the orbit to the zygomatic process of the malar bone. The flap was then dissected up and turned back. After removing the middle incisor tooth, the bone was cut with strong pliers through the palate process, near its fellow, then through the internal and external orbital angles, leaving the orbital plate, also the palate process of the palate bone. All bony connections having been severed, the tumor was grasped with the lion-jawed forceps, depressed, slightly twisted and pulled forward, and readily removed. Some slight adhesions being divided with the knife.

A good deal of blood was lost from the severed arteries of the lip and cheek, but the bleeding was controlled by pressure from the fingers of the assistants. No bleeding followed the removal of the tumor. A white shining membrane was left at the bottom of the cavity, and when the flaps were adjusted no ligature was required. Every one was surprised at the slight loss of blood, and that neither ligature nor tension was necessary to control the bleeding.

The cavity was filled with pieces of lint fastened to a silk ligature to assist in their removal. The incision was accurately adjusted with silver sutures; a cloth wet with cold water was laid over the face and the man placed in bed. As soon as the effects of the chloroform wore off gr. ss. of morphia was given hyperdermically.

The man slept well through the night, and was freer from pain than he had been for months. He made a rapid recovery and was discharged June 8th with the wound en tirely closed.

A letter received from him by Dr. Taliaferro, Superintendent of the Hospital, dated July 4th, says that he is rapidly recovering his general health and hopes soon to be as well as ever.—*Virginia Clinical Record.*

